# Supplementary material for: Modelling the effects of adult emergence on the surveillance and age distribution of medically important mosquitoes
Source: PLoS Comput Biol. 2025 Aug 18;21(8):e1013035. doi: 10.1371/journal.pcbi.1013035 (PMC12373278; doi:10.1371/journal.pcbi.1013035)
Supplement: S1 Text — (PDF) [file pcbi.1013035.s001.pdf]

# S1 Text. Compartmental mosquito model supplementary methods

Seasonality in the mosquito population dynamics is achieved by varying the carrying capacity. The number of eggs laid per day, $\beta_t$, is calculated as,

$$\beta_t = \frac{\beta_l}{\mu_t}(e^{\frac{\mu_t}{f_{v,t}}} - 1)\mu_t \frac{e^{\frac{\mu_t}{f_{v,t}}}}{(1 - e^{\frac{\mu_t}{f_{v,t}}})}, \tag{S1}$$

where $\beta_l$ is the maximum number of eggs per female mosquito per oviposition cycle, $\mu$ is the per-capita adult mosquito mortality rate, $f_v$ and is the per-capita feeding rate. The carrying capacity is calculated thus,

$$\begin{aligned}
\omega_s &= \gamma\frac{\mu_l}{\mu_e} - \frac{d_{el}}{d_l} + (\gamma - 1)\mu_l d_{el}, \\
\omega_t &= -0.5\omega_s + \sqrt{0.25\omega_s^2 + \frac{0.5\gamma\beta_t\mu_l d_{el}}{\mu_e\mu_0 d_l(1 + d_{pl}\mu_p)}}, \\
K_0 &= \frac{M_0 2 d_l \mu_0(1 + d_{pl}\mu_p)\gamma(\omega + 1)}{\frac{\omega_t}{\mu_l d_{el}} - \frac{1}{\mu_l d_l} - 1}, \\
K(t) &= K_0\frac{r(t)}{\bar{r}},
\end{aligned} \tag{S2}$$

where $\gamma = 13.25$ is the effect of density dependence on late instars relative to early instars; $\mu_e = 0.0338$, $\mu_l = 0.0348$ and $\mu_p = 0.249$ per-day are the per-capita mortality rates of early stage larvae, late stage larvae and pupae respectively; $\mu_0 = 0.132$ per day is the adult mosquito mortality rate; $d_{el} = 6.64$, $d_l = 3.72$ and $d_p = 0.643$ days are the development times of early stage larvae, late stage larvae and pupae respectively; $M_0 = 200$ is the initial mosquito density. Seasonality in the mosquito carrying capacity is modelled according to a Fourier series,

$$\begin{aligned}
r(t) &= \max(g_0 + \sum_{i=1}^{3} g_i cos(2\pi i\frac{t}{365}) + \sum_{i=1}^{3} h_i sin(2\pi i\frac{t}{365}), 0.1), \\
\bar{r} &= \sum_{j=1}^{365} r(j),
\end{aligned} \tag{S3}$$

where $g_0 =$, $g_1$, $g_2$, $g_3$, $h_1$, $h_2$ and $h_3$ were previously estimated by fitting to rainfall data from the Cascades region of Burkina Faso. For the perennial simulations $r(t)$ was fixed at 1.

The ITN model follows that devised previously by Le Menach [1] and parameterised in Griffin [2]. Briefly, the probability of surviving one feeding or resting cycle are calculated as,

$$\begin{aligned}
p_{10} &= e^{-\mu_0\tau_1}, \\
p_2 &= e^{-\mu_0\tau_2},
\end{aligned} \tag{S4}$$

respectively, where $\tau_1 = 0.69$ days is the duration of host seeking behaviour and $\tau_2 = 2.31$ days is the duration of resting behaviour. $\mu$ and $f_v$ are calculated according to ITN population-level usage the previous night (coverage). Once ITNs are distributed we assume people continue to use them throughout the simulation. Depending on the coverage level people can either either sleep under or not sleep under a net, though all people living in the area will be receive some protection from the community impact of

ITNs. The probability a mosquito bites and survives is $W_{nc} = 1$ for those not protected by an ITN and $W_{c,t} = 1 - \phi_B + \phi_B S_{ITN,t}$, where $\phi_B = 0.89$ is the proportion of mosquitoes biting in bed and $S_{ITN,t} = 1$ if there are no ITNs and $S_{ITN,t} = 1 - D_{ITN,t} - R_{ITN,t}$ if there are ITNs. $D_{ITN,t}$ is the probability a blood-meal seeking mosquito dies due to the ITN and $R_{ITN,t}$ is the probability a mosquito is repelled due to the ITN. The probability a mosquito is repelled during a feeding attempt is $Z_{nc} = 0$ for people without ITNs and $Z_{c,t} = \phi_B R_{ITN,t}$ for those using an ITN. The average probability of repellency or successful feeding, $\bar{Z}$ and $\bar{W}$ respectively, for a feeding attempt is,

$$\begin{aligned} Z_t &= (Z_{nc}(1 - c_{ITN}) + Z_{c,t} c_{ITN}), \\ W_t &= (W_{nc}(1 - c_{ITN}) + W_{c,t} c_{ITN}), \\ \bar{Z}_t &= Q_0 Z_t, \\ \bar{W}_t &= 1 - Q_0 + Q_0 W, \end{aligned} \tag{S5}$$

where $c_{ITN}$ is the ITN coverage and $Q_O = 0.92$ is the proportion of anthropophagy. The ITNs efficacy decays with time,

$$\begin{aligned} D_{ITN,t} &= D_{ITN0} e^{t - t_{dist} l_{ITN}}, \\ R_{ITN,t} &= R_{ITN1} + (R_{ITN0} - R_{ITN1}) e^{t - t_{dist} l_{ITN}}, \end{aligned} \tag{S6}$$

where $t_{dist}$ is the ITN distribution time, $D_{ITN0} = 0.41$ is the initial proportion of mosquitoes that die due to the ITN, $l_{ITN} = \frac{log(2)}{2.64365}$ per day is the rate at which the ITN efficacy decays, $R_{ITN1} = 0.24$ is the minimum proportion of mosquitoes repelled by an ITN and $R_{ITN0} = 0.56$ is the initial proportion of mosquitoes repelled by an ITN. The probabilities of surviving one feeding or resting cycle are updated due to the ITNs,

$$\begin{aligned} p_{1,t} &< -\frac{\bar{W}_t p_{10}}{1 - \bar{Z}_t p_{10}}, \\ f_{v,t} &= \frac{1}{\frac{\tau_1}{(1-\bar{Z})} + \tau_2}, \\ \mu_t &= -f_{v,t} log(p_{1,t} p_2). \end{aligned} \tag{S7}$$

# References


1. Le Menach, A. *et al.* An elaborated feeding cycle model for reductions in vectorial capacity of night-biting mosquitoes by insecticide-treated nets. *Malaria journal* **6,** 1–12 (2007).

2. Griffin, J. T. *et al.* Reducing *Plasmodium falciparum* malaria transmission in Africa: a model-based evaluation of intervention strategies. *PLoS Medicine* **7,** e1000324 (Aug. 2010).
