# Supplementary figures and images for: Modelling the effects of adult emergence on the surveillance and age distribution of medically important mosquitoes
Source: PLoS Comput Biol. 2025 Aug 18;21(8):e1013035. doi: 10.1371/journal.pcbi.1013035 (PMC12373278; doi:10.1371/journal.pcbi.1013035)

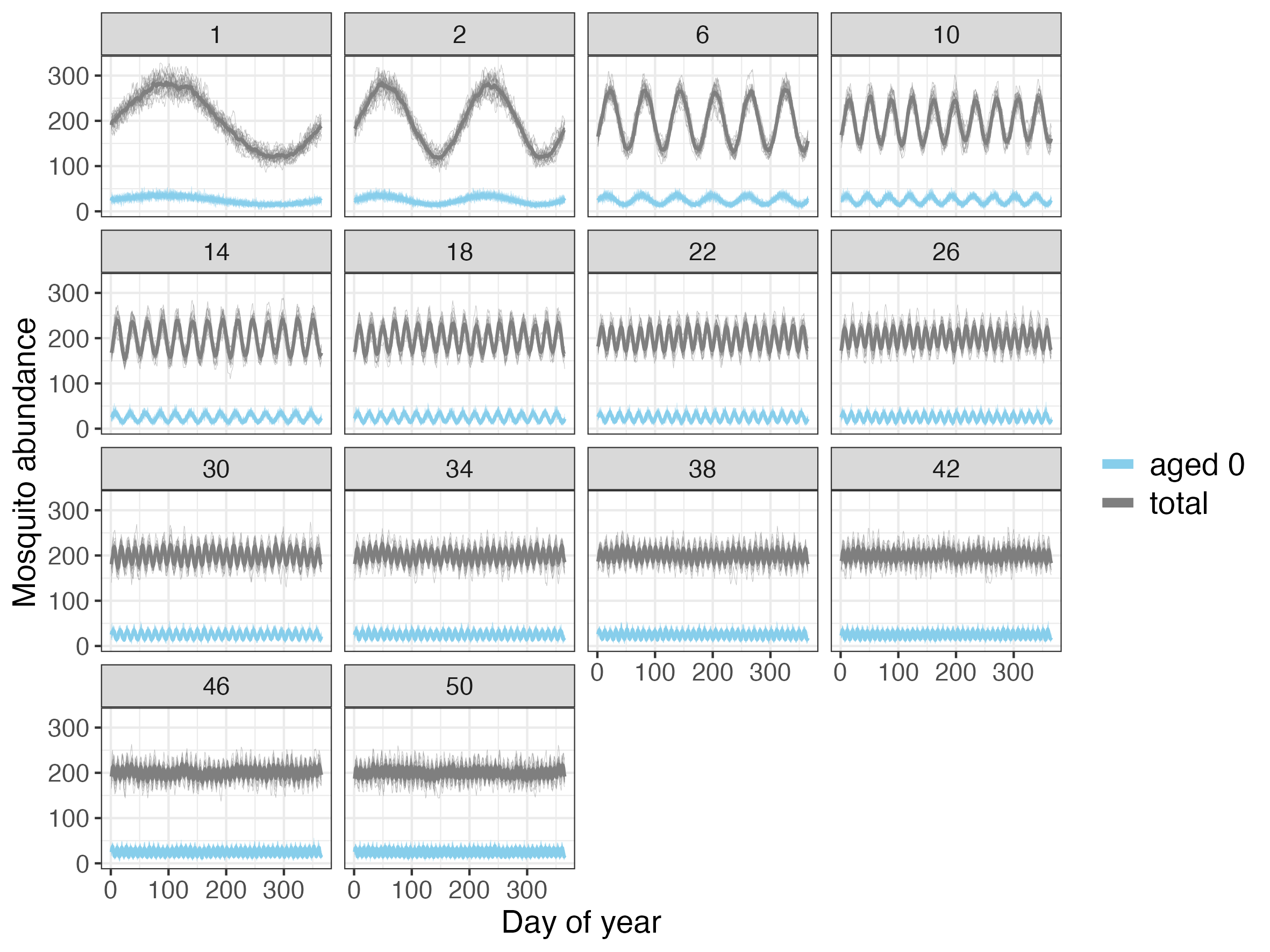

Supplement: S1 Fig — Facet show the period of the sine wave per year (multiplied by 365; 365κ). The amplitude of adult mosquito emergence was assumed to be constant at 10 mosquitoes, which is approximately 40% of the mean emergence rate mean (100ηo). (TIFF) [file pcbi.1013035.s002.tiff]

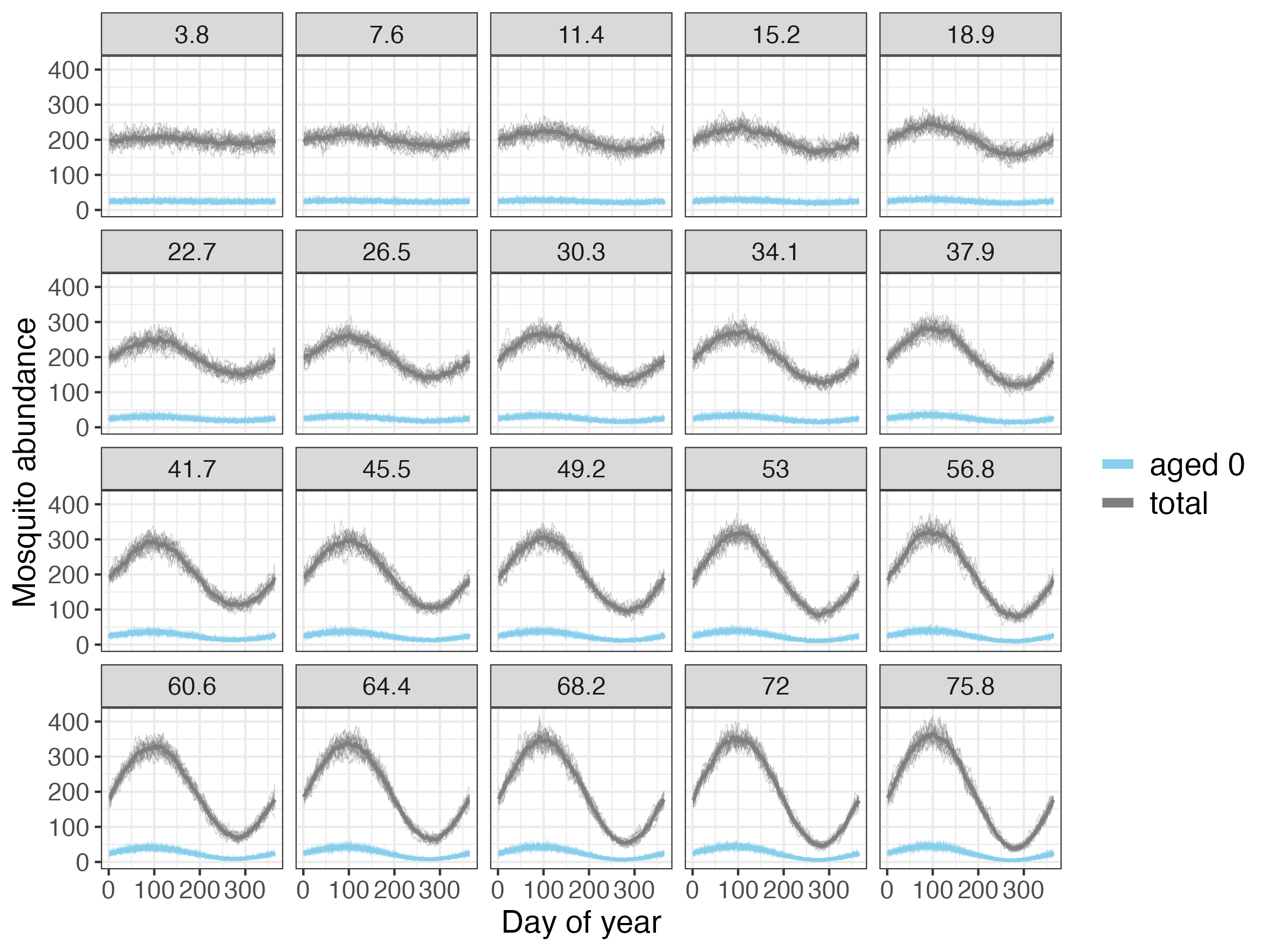

Supplement: S2 Fig — Facet show the amplitude as a percent of the mean emergence rate mean (100ηo). The frequency of adult mosquito emergence was assumed to be constant with a single peak per year. (TIFF) [file pcbi.1013035.s003.tiff]

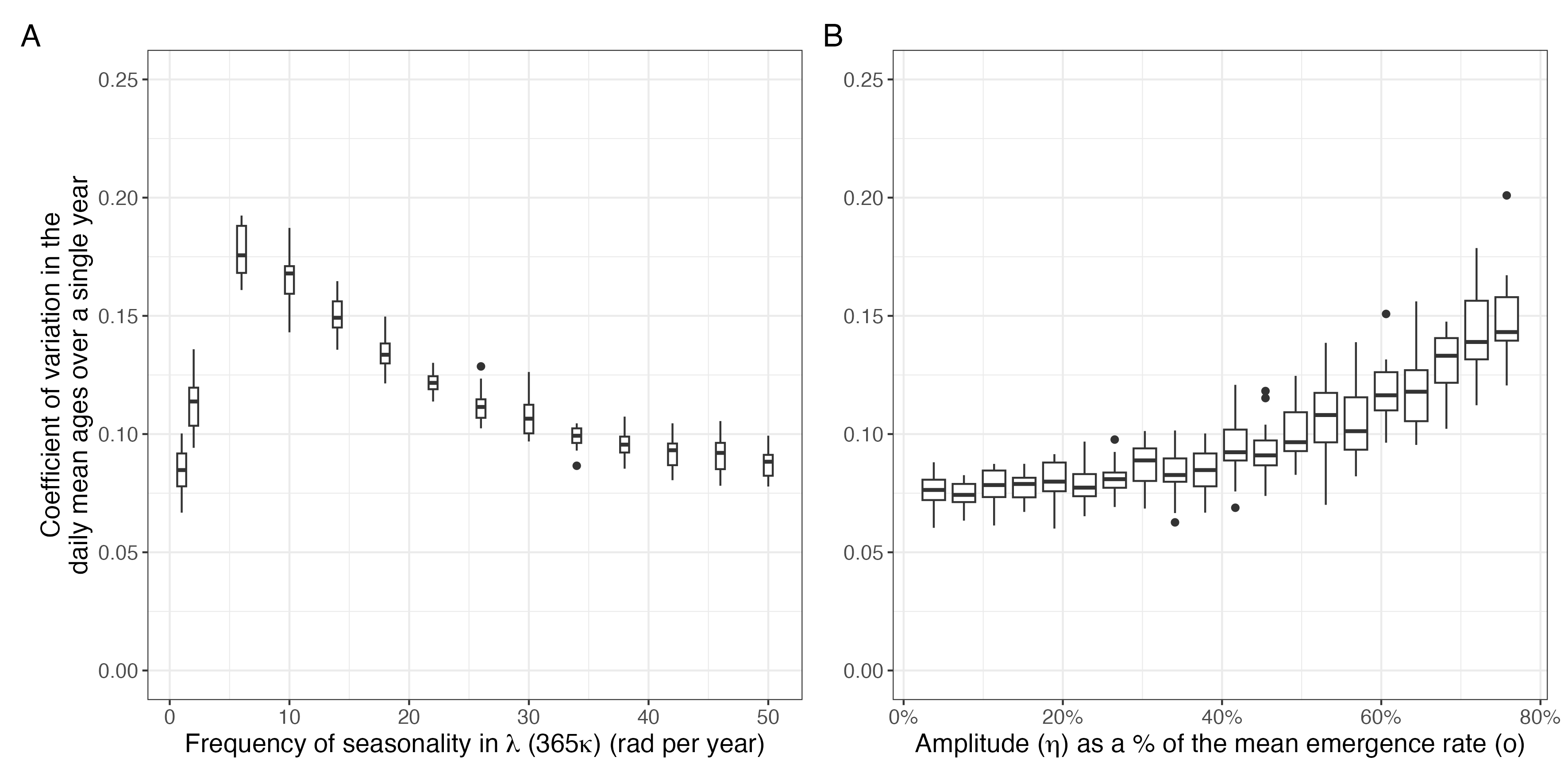

Supplement: S3 Fig — (A) Mosquito emergence was simulated to fluctuate according to a sine wave with the number of periods per year. The mean mosquito emergence was assumed to be equal to the initial mosquito population size multiplied by the daily probability of mortality. An amplitude of 10 is assumed, which is approximately 40% of the mean of the mean emergence rate (o¯). (B) The temporal variability in the daily mean mosquito age given different amplitude fluctuations in the mean mosquito emergence rate and a frequency (365κ) of 1 radians per year. For (A) and (B) boxplots show the variability between different simulations with the ggplot2 default parameters (the box shows the 25%, 50% and 75% quantiles). (TIFF) [file pcbi.1013035.s004.tiff]

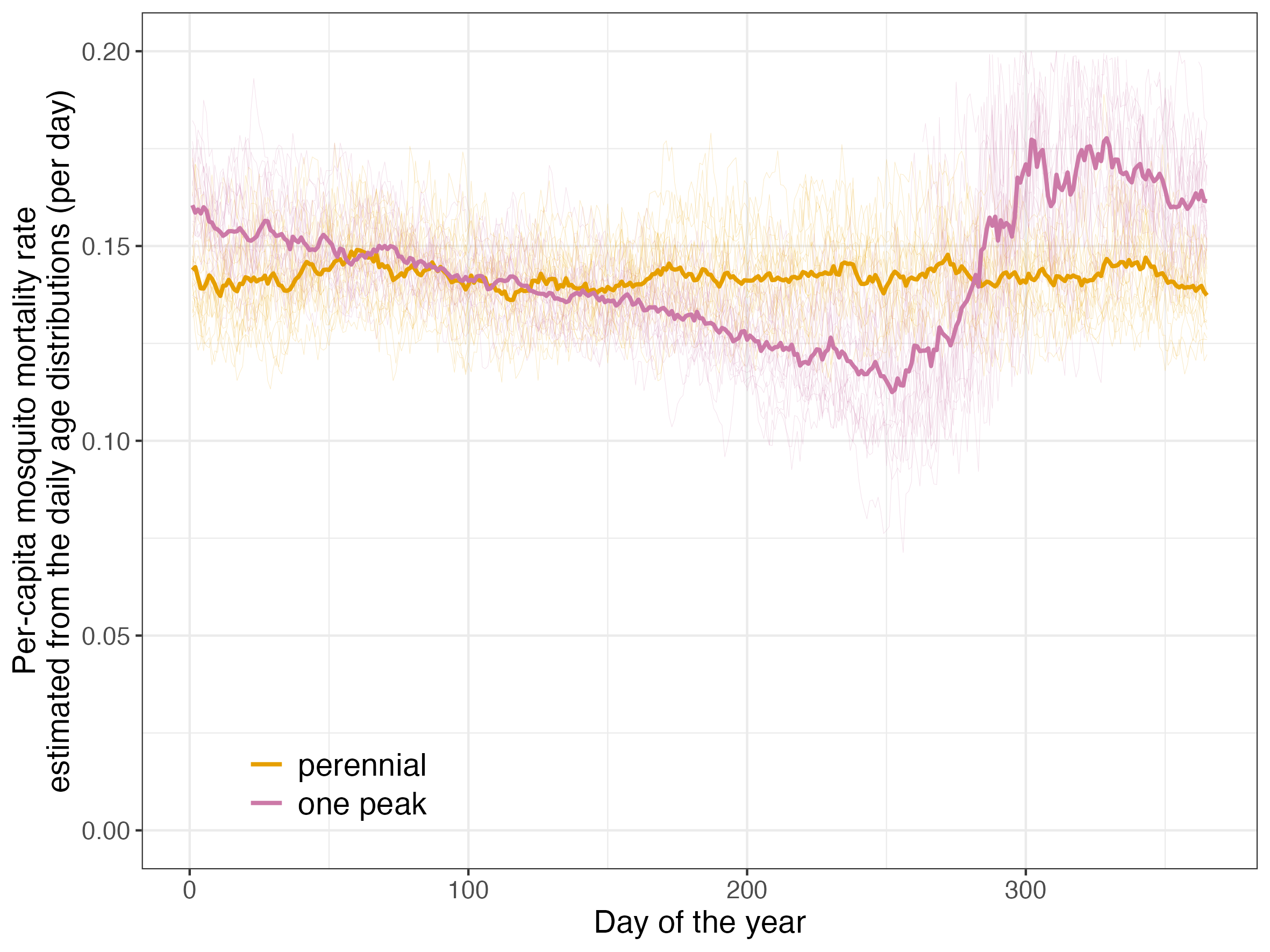

Supplement: S4 Fig — The pink/purple lines show the results where the mosquito populations is simulated with an adult mosquito emergence rate that follows a sine wave with a single period per year and the yellow lines show those with perennial mosquito population dynamics. The thin lines show the results from a single simulation and the thicker lines show the mean values of all the simulations. (TIFF) [file pcbi.1013035.s005.tiff]

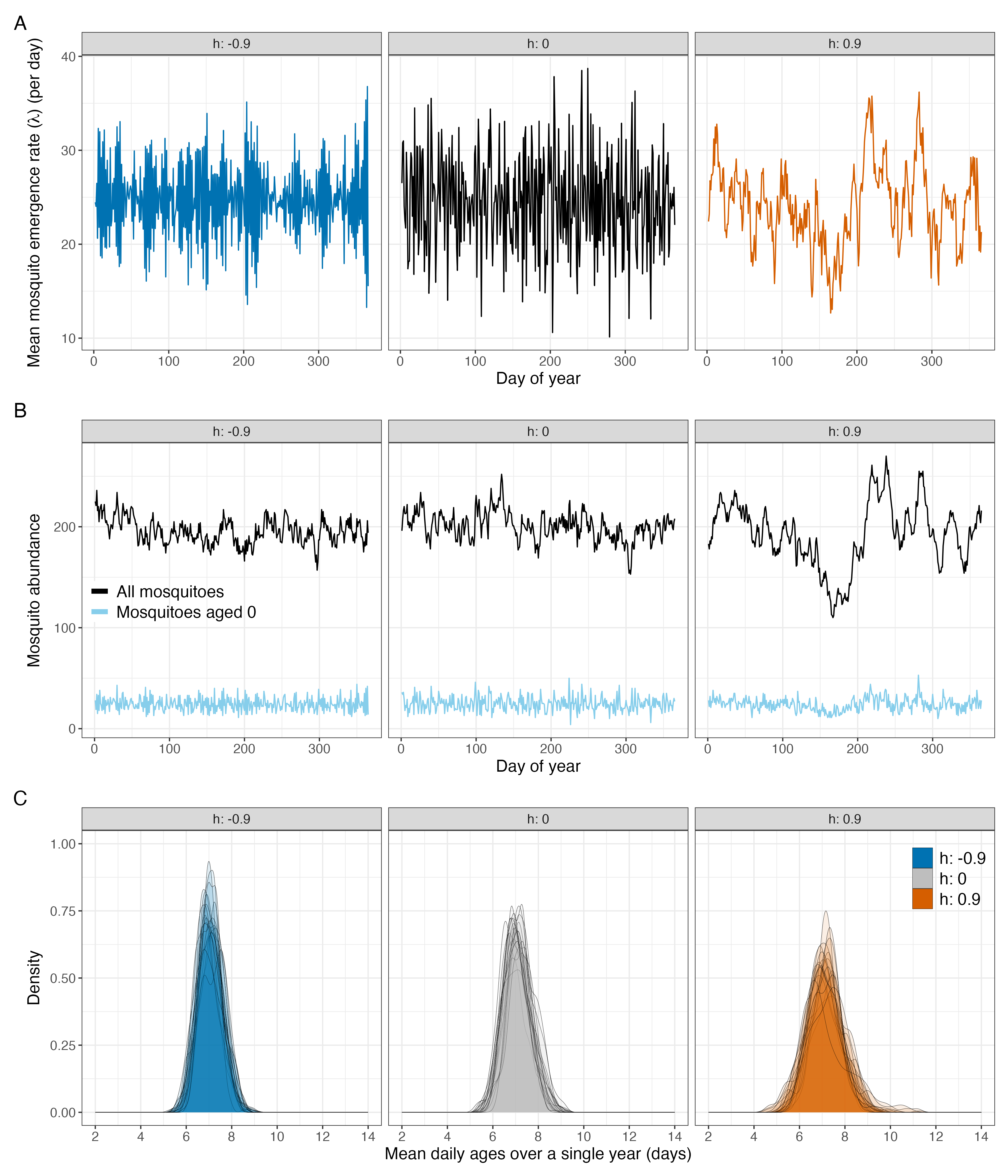

Supplement: S5 Fig — (A) The theoretical noise in the mean mosquito emergence rate (λt) for a single year for a single simulation, given a lag-1 autoregressive model with the same mean and standard deviation but different levels of autocorrelation (h). (B) The temporal dynamics of mosquito abundance (black) and newly emerged mosquitoes (between 0 and 1 day old) corresponding to the λt values in A. (C) The distribution of the simulated mean daily ages over a single year given different levels of autocorrelation. Temporal variability in the mean ages is presented as a density because different simulations with the same autocorrelation parameters do not necessarily shows the same trends at the same time. The density was estimated from the the frequencies of the mosquito ages using a Gaussian kernel. For all plots the mean of the mean emergence rate (o) was assumed to be (26.4) and the standard deviation (σ) 5 in the lag-1 autoregressive model. (TIFF) [file pcbi.1013035.s006.tiff]

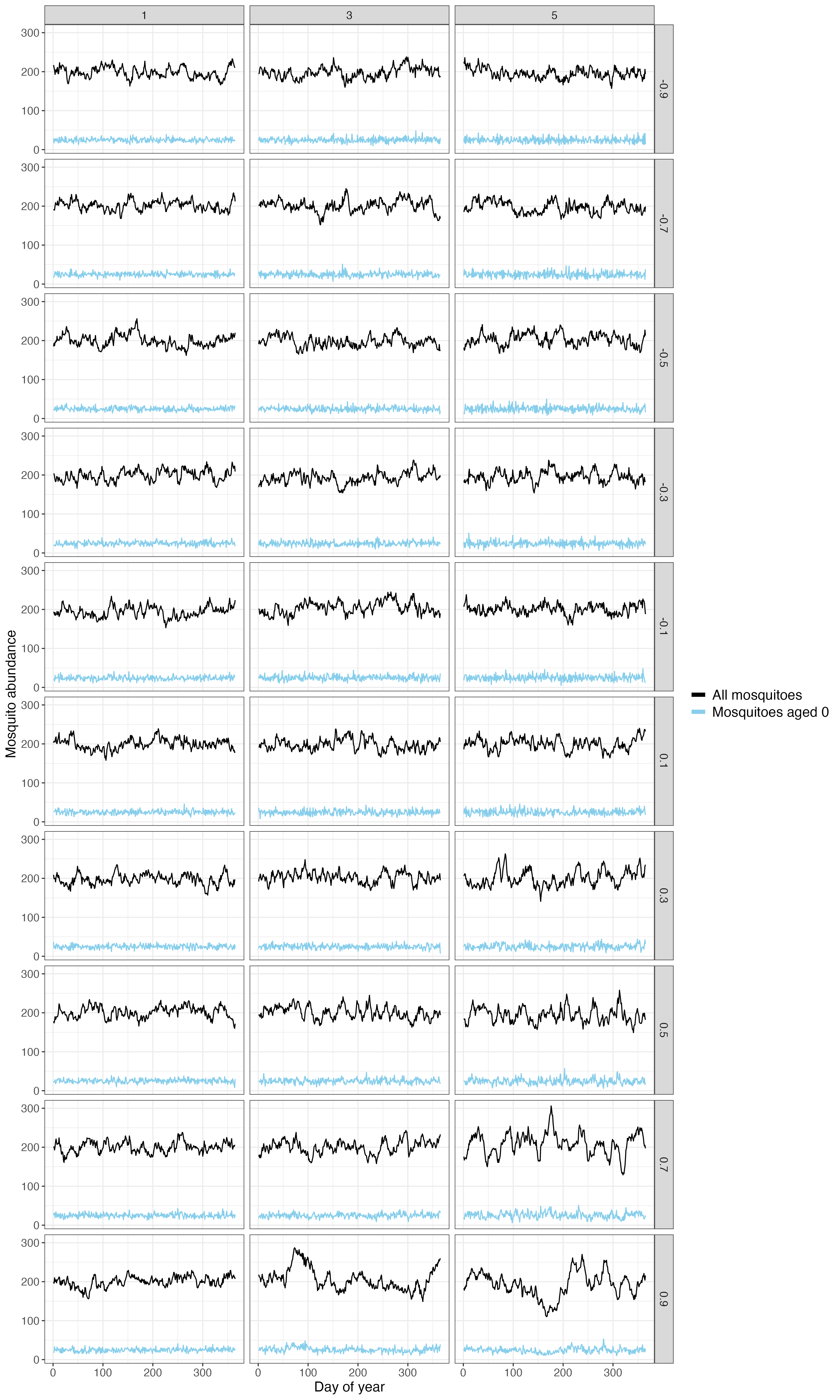

Supplement: S6 Fig — Facet columns show the standard deviation (σ) and facet rows show the autocorrelation (h). Values are shown for a single simulation. (TIFF) [file pcbi.1013035.s007.tiff]

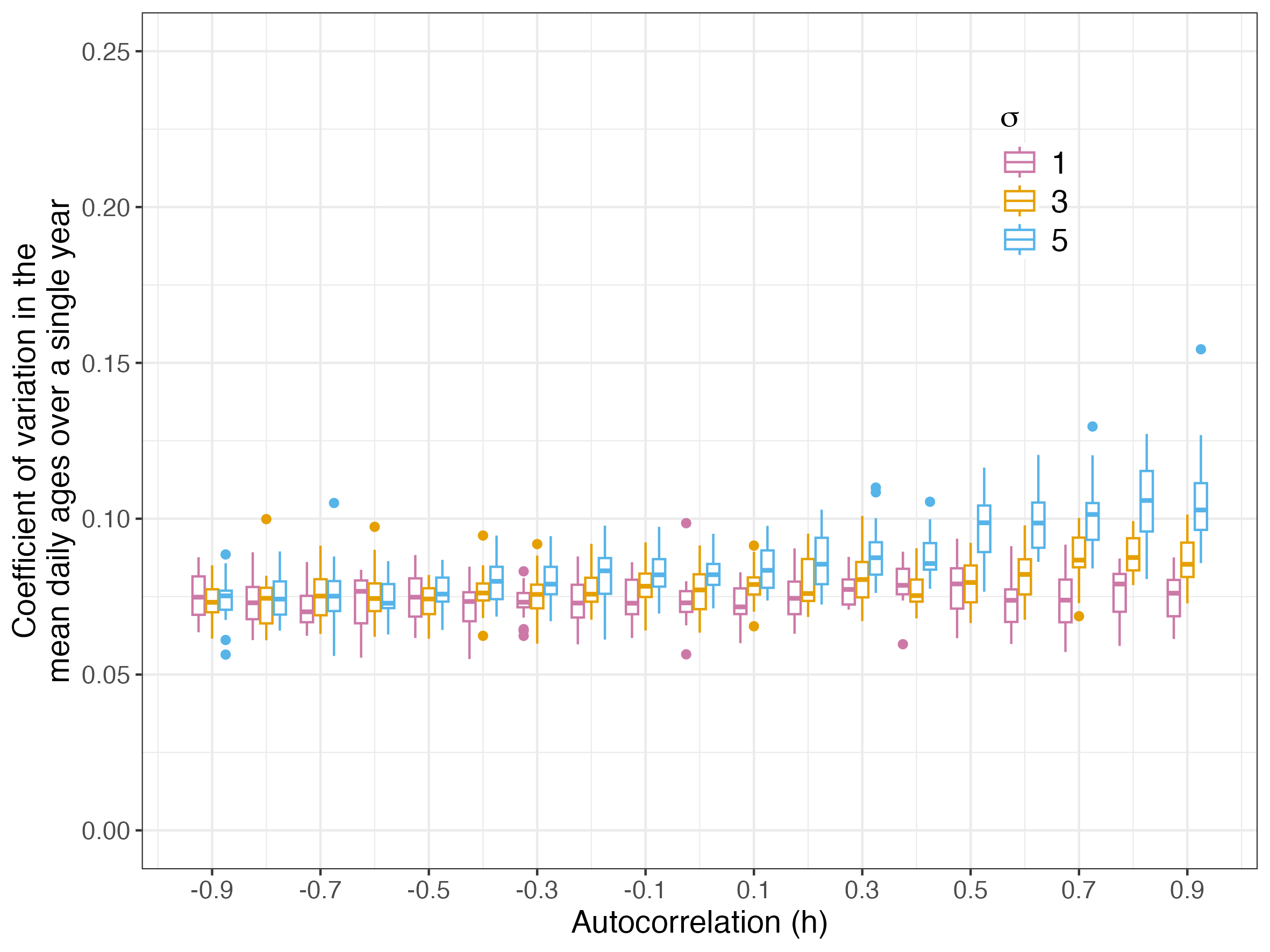

Supplement: S7 Fig — Boxplots show the variability between different simulations with the ggplot2 default parameters (the box shows the 25%, 50% and 75% quantiles). (TIFF) [file pcbi.1013035.s008.tiff]

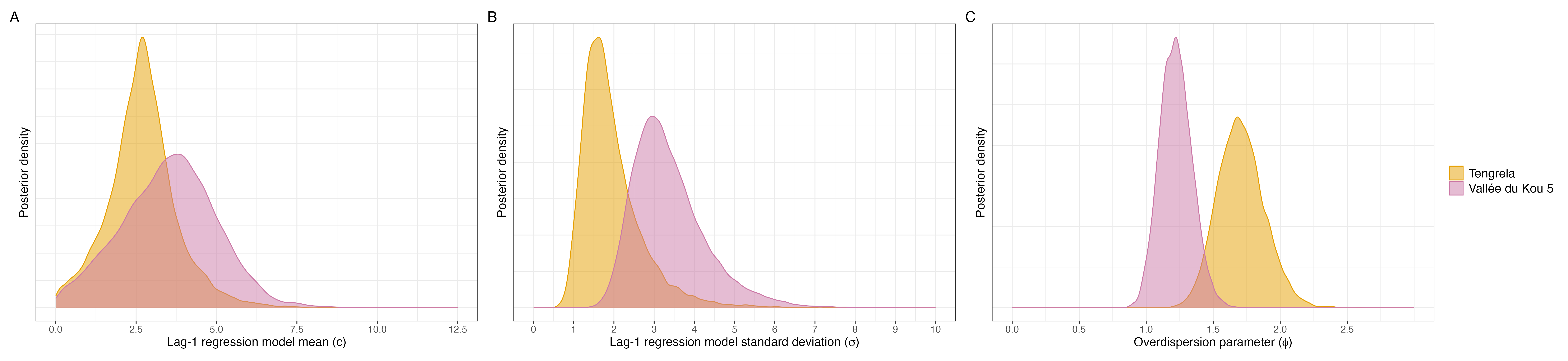

Supplement: S8 Fig — (TIFF) [file pcbi.1013035.s009.tiff]
